# Docking simulation between HIV peptidase inhibitors and *Trypanosoma cruzi aspartyl peptidase*

**DOI:** 10.1186/s13104-018-3927-z

**Published:** 2018-11-21

**Authors:** Vanessa V. S. Castilho, Keyla C. S. Gonçalves, Karina M. Rebello, Luiz P. R. Baptista, Leandro S. Sangenito, Helena L. C. Santos, Marta H. Branquinha, André L. S. Santos, Rubem F. S. Menna-Barreto, Ana C. Guimarães, Claudia M. d’Avila-Levy

**Affiliations:** 1 Laboratório de Genômica Funcional e Bioinformática, Instituto Oswaldo Cruz, Fundação Oswaldo Cruz, Rio de Janeiro, Brazil; 2 Laboratório de Estudos Integrados em Protozoologia, Instituto Oswaldo Cruz, Fundação Oswaldo Cruz, Rio de Janeiro, Brazil; 3 Laboratório de Estudos Avançados de Microrganismos Emergentes e Resistentes (LEAMER), Instituto de Microbiologia Paulo de Góes, Universidade Federal do Rio de Janeiro, Rio de Janeiro, Brazil; 4 Laboratório de Biologia Celular, Instituto Oswaldo Cruz, Fundação Oswaldo Cruz, Rio de Janeiro, Brazil; 5 Present Address: de Duve Institute, Université Catholique de Louvain, Brussels, Belgium

**Keywords:** Aspartic peptidase, Chagas’ disease, Chemotherapy, Neglected tropical diseases, Drug-repurposing

## Abstract

**Objective:**

The low investment in research, diagnosis and treatment are factors that contribute to the continuity of Chagas’ disease as a neglected tropical diseases (NTDs). In this context, the repositioning of drugs represents a useful strategy, in the search for new chemotherapeutic approaches for NTDs. HIV aspartic peptidase inhibitors (HIV IPs) are good candidates for drug repurposing. Here, we modeled the three dimensional structure of an aspartyl peptidase of *Trypanosoma cruzi*, the causative agent of Chagas’ disease, aligned it to the HIV aspartyl peptidase and performed docking binding assays with the HIV PIs.

**Results:**

The 3D structure confirmed the presence of acid aspartic residues, which are critical to enzyme activity. The docking experiment revealed that HIV IPs bind to the active site of the enzyme, being ritonavir and lopinavir the ones with greater affinity. Benznidazole presented the worst binding affinity, this drug is currently used in Chagas’ disease treatment and was included as negative control. These results together with previous data on the trypanocidal effect of the HIV PIs support the hypothesis that a *T. cruzi* aspartyl peptidase can be the intracellular target of these inhibitors. However, the direct demonstration of the inhibition of *T. cruzi* aspartyl peptidase activity by HIV PIs is still a goal to be persuaded.

**Electronic supplementary material:**

The online version of this article (10.1186/s13104-018-3927-z) contains supplementary material, which is available to authorized users.

## Introduction

Chagas disease, caused by the kinetoplastid parasite *Trypanosoma cruzi*, affects 8 million people worldwide and about 10,000 people die of complications linked to the disease [1]. The classical contamination route involves a triatomine vector bite, usually associated with poor habitation conditions in Latin America. However, human immigration associated with blood transfusions contributed to the spread of the disease to North America, Europe and some Western Pacific countries. This change in epidemiological scenario led to an increased report of co-infected patients with Chagas disease and acquired immune deficiency syndrome (AIDS) [2–4]. Even tough more than a century has passed since the discovery of Chagas disease, the drug repertoire is still based on benznidazole and nifurtimox, which are compounds with severe side effects, questionable specificity and efficacy [5, 6]. Although some promising drug candidates are under development, such as ravuconazole, posoconazole and fexinidazole [7–9], the questionable economic return of investing in drug development for a disease mainly associated with poverty discourages pharmaceutical companies to search for new chemotherapeutics against this parasitic illness [5].

In this context, drug-repurposing strategy has the potential to facilitate an effective chemotherapeutic approach [10, 11]. The marked improvement in the life expectancy of AIDS sufferers after the incorporation of HIV aspartic peptidase inhibitors (HIV IPs) into the drug cocktail, the so-called highly active anti-retroviral therapy (HAART), was due to reduction in viremia, recovery of the immune response and a direct action on opportunistic pathogens [reviewed in 3]. The last effect has been extensively demonstrated in *Trypanosoma cruzi* by our research group [3, 12–14] and others [15], although the mode of action and intracellular target of the compounds are still unknown and have been a matter of extensive research [3, 14, 16, 17]. Here, we identified in the genome of *T. cruzi*, CL-Brener strain, a homologue of the HIV retroviral peptidase and selected a crystalized protein for generating a three dimensional model, which was aligned with HIV aspartyl peptidase. Then, we performed docking experiments between the modeled 3D protein and HIV PIs. We have also included pepstatin A and benznidazole as a reference for positive and negative controls, respectively, since the former is a classical aspartyl peptidase inhibitor [18] and the latter a classical drug used in Chagas’ disease treatment with unrelated mechanism of action and binding site [5].

## Main text

### Methods

#### Comparative modeling

The crystal structures of the retroviral aspartic peptidase domain of DNA-damage inducible proteins (DDI-1 like) from *T. cruzi* were not available in the Protein Data Bank (PDB) and a comparative modeling was performed to predict their 3D structures. The amino acid sequences of these target proteins of *T. cruzi* (strain CL brenner; entries Tc00.1047053510155.40 and Tc00.1047053511585.40 from TriTrypdb) were submitted to BLASTp search through the PDB to retrieve similar proteins with available 3D structure. The crystal structures selected as templates were the retroviral peptidase-like domains of the DDI-1 like proteins from *Homo sapiens* (PDB ID 4RGH, sequence identity 43% for Tc00.1047053510155.40 and 44% for Tc00.1047053511585.40) and *Saccharomyces cerevisiae* (PDB ID 4Z2Z, sequence identity 41% for both *T. cruzi* sequences). The comparative modeling was carried out using the selected templates and the auto-model module from MODELLER 9v16 [19]. The two final homodimeric models of aspartyl peptidase domains (presenting the residues 227-353/354) of the target proteins of *T. cruzi* were selected based on the DOPE scoring function. These models were called TcRP-A and TcRP-B for the retroviral aspartic peptidase domain of Tc00.1047053510155.40 and Tc00.1047053511585.40, respectively. An energy minimization using the Amber ff14SB force field through UCSF CHIMERA interface [20] was performed to improve the overall structure geometry of the both TcRP-A and TcRP-B models. The stereochemical quality of the two refined models was evaluated using the ERRAT and VERIFY 3D from SAVES server (http://services.mbi.ucla.edu/SAVES/) and MOLPROBITY (http://molprobity.biochem.duke.edu/).

A multiple sequence alignment was performed using the ClustalW through Multiple Sequence Viewer of Maestro (Schrödinger Suite) in order to evaluate the sequence identity and similarity between the aspartyl peptidase domain of DDI-1 like proteins from *T. cruzi* and the HIV-1 peptidase. The superposition of the 3D structures of the refined models and the HIV-1 peptidase (PDB ID 3OXC) and the root mean square deviation (RMSD) between them were obtained with the “super” command in Pymol v1.8.2.1 (http://www.pymol.org/). Further alignments and comparisons with these structures were carried out with the TM-align algorithm (http://zhanglab.ccmb.med.umich.edu/TM-align/) [21].

#### Molecular docking

In order to predict the inhibitory potential and binding modes of HIV-PIs towards the aspartyl peptidase domain of DDI-1 like protein of *T. cruzi*, molecular docking assays were performed with the constructed TcRP-A and TcRP-B models. Besides that, pepstatin A, a classical aspartyl peptidase inhibitor [18], was included as a positive control, while benznidazole was used as a negative control, since it has an unrelated mode of action. These ligands were retrieved in the sdf format from PubChem and prepared by the ligand preparation module (LigPrep) of Maestro. As an outcome, ionization states at a pH of 3.5 and 5.0 and tautomers were generated, the specified chiral centers were retained, and the resulted molecules were energetically minimized using the OPLS-2005 force field.

Both *T. cruzi* protein models were prepared using the protein preparation wizard of Maestro, in which hydrogen atoms were added, and protonation states were determined at pH 3.5 with PROPKA. Grids were generated around the potential active site of the two prepared protein models using the Receptor Grid Generation module of Glide, also from Maestro. The grid box was set to have 35Å of edge with coordinates x = − 220.53, y = 32.3, and z = 16.96 for TcRP-A and x = 66.35, y = − 8.35, and z = 13.47 for TcRP-B, both coordinates were determined using the Asp248A, Asp248B and Arg279B (one of the potential substrate binding residues) as centroids. Following ligand and protein preparation, as well as grid generation, docking simulations were performed with the extra precision (XP) protocol from Glide (GlideXP) (version 6.7) [22]. The potential inhibitors were docked in the potential active site of TcRP-A and TcRP-B domains. The inhibitors were ranked based on their docking scores.

In a similar manner, the HIV protein (PDB ID 3OXC) was prepared with protein preparation wizard at pH 5.0, once the optimal pH range described for HIV-1 peptidase activity is 4–6 in contrast to eukaryotic aspartyl peptidases, which have, in general, optimal pH range of 2–4 [23]. The grid box was centered on the crystal structure of inhibitor saquinavir complexed with the studied HIV protein (x = 5.04, y = − 2.74, and z = 14.8) and had an edge of 35 Å. Besides the redocking of saquinavir inhibitor with XP protocol from Glide, all other well know HIV inhibitors were also docked, and benznidazole compound was used as negative control.

### Results and discussion

To perform structural analysis and molecular docking studies, two 3D structures of the aspartyl peptidase domain of DDI-1 like proteins from *T. cruzi* were constructed through comparative modeling. For this purpose, the homodimeric retroviral aspartyl peptidase domains of DDI-1 like proteins from humans (PDB ID 4RGH) and yeast (PDB ID 4Z2Z) were used as templates. The models TcRP-A and TcRP-B were selected by considering their DOPE scores of − 29,695.480 and − 29,901.357, the lowest among the set of models generated for Tc00.1047053510155.40 and Tc00.1047053511585.40, respectively. The Ramachandran plot of the two final refined models indicated that 96.4% and 96% of the amino acids for TcRP-A and TcRP-B, respectively, are in favored regions, and 99.2% (in both cases) are in allowed regions (data not shown). The Errat overall quality factor for TcRP-A was 90.783 and 87.037 for TcRP-B. The Verify 3D server estimated that 95.31 and 97.66% of the residues of TcRP-A and TcRP-B, respectively, had an averaged 3D-1D score ≥ 0.2. These results indicate that the two refined models have good quality and are reliable for carrying out further computational analysis.

**Fig. 1:**
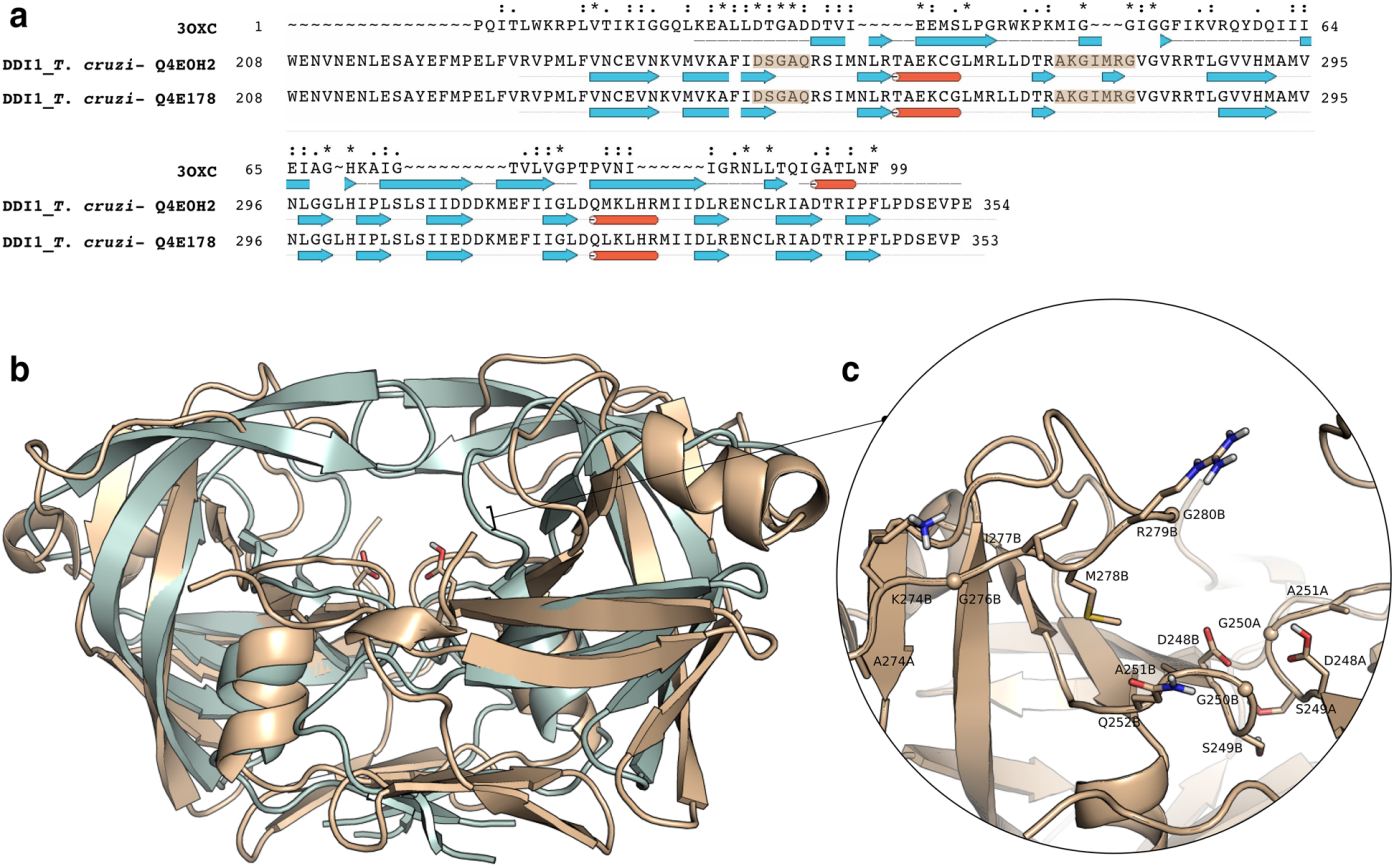
Multiple sequence alignment and structural superposition of retroviral aspartyl peptidase domain of from *T. cruzi* and HIV-1. **a** Multiple sequence alignment of the retroviral aspartyl peptidase domains of *T. cruzi* and the HIV-1 peptidase (TcRP-A: UNIPROT ID Q4E0H2, TcRP-B: UNIPROT ID Q4E178, HIV-1 peptidase: PDB ID 3OXC). The active site residues from the *T. cruzi* domains are highlighted in light orange. Above the aligned sequences, identical sequences are indicated by an asterisk (*), residues with strongly similar properties are indicated by a colon (:), and residues with weakly similar properties are indicated by a period (.). Below the aligned sequences, blue arrows indicate β-sheet, while the red cylinder indicates α helices in the secondary structure of the protein. **b** Structural superposition of the 3D structures of HIV-1 peptidase (PDB ID 3OXC, in grey) and the TcRP-B model (in brown) in cartoon representation. The catalytic aspartates (Asp248A and B) are represented in sticks. **c** Surface and cartoon representation of the active site of TcRP-B model. The amino acids located in the active site are represented in sticks and the glycine residues are represented in spheres. TcRP-A model presented similar results (data not shown)

The presence of a retroviral aspartyl peptidase domain in the DDI-1 like proteins of *T. cruzi* suggests the structural similarity between the same domains in proteins of different organisms. In order to visualize these similarities, a comparison between the primary, secondary and 3D structures of the HIV-1 peptidase domain (PDB ID: 3OXC) and the generated models were performed (Fig. 1). The sequence alignment of TcRP-A/B and HIV-1 peptidase showed an overall identity of 12% and a similarity of 28% (Fig. 1a). Despite the low identity, the superimposition of the 3D structures confirms the similarity between these domains with an RMSD of 2.67 Å for TcRP-A and 2.98 Å for TcRP-B in relation to HIV-1 peptidase (Fig. 1b). The sequence-order independent structure comparisons using the TM-align emphasize these structural similarities with a TM-score value of ~ 0.71 for both models towards HIV-1 peptidase, indicating that these domains have the same fold. The potential active site of the retroviral aspartyl peptidase domain of *T. cruzi* (TcRP-B model) is shown in Fig. 1c. The models for the retroviral aspartyl peptidase domain were generated as homodimers with the active sites containing residues from both subunits A and B such as the catalytic aspartic peptidases (Asp238A and B). Besides other active site residues, the potential substrate-binding loop (residues Ala274-Gly280) described for retroviral aspartyl peptidase domain of yeast [24] are also indicated in Fig. 1c.

**Fig. 2:**
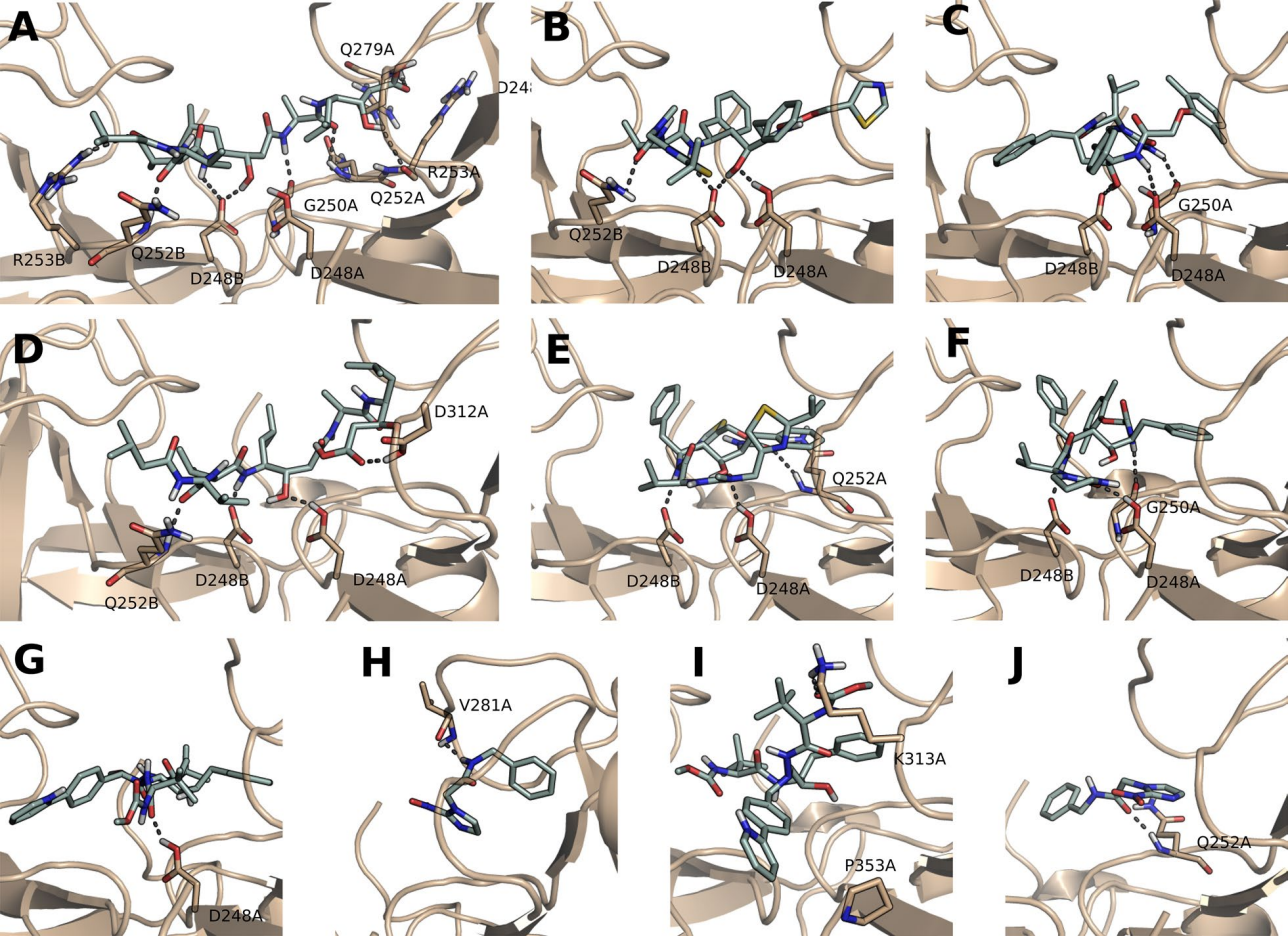
Intermolecular interactions between the two *T. cruzi* aspartyl peptidase domains and the inhibitor compounds. We selected the three compounds with higher hits (**A**–**F**) and the two with lower hits in docking simulations (**G**–**J**). Hydrogen bond interactions observed between TcRP-A (**A**–**C**; **G**, **H**) and TcRP-B (**D**–**F**; **I**, **J**), and the three compounds that gave the higher hits after the molecular docking simulation: pepstatin A (**A**, **D**), ritonavir (**B**, **E**) and lopinavir (**C**, **F**); and the two compounds with lower hits: atazanavir (**G**, **I**) and benznidazole (**H**, **J**)

After the design and validation of the 3D models, we performed molecular docking simulations aiming to predict inhibitory potential (docking scores) and the intermolecular interactions of the HIV aspartic peptidase inhibitors towards the active sites of the two modeled retroviral aspartyl peptidase domains (TcRP-A and TcRP-B) of the DDI-1 like proteins from *T. cruzi*. The ligands were classified based on their docking scores, as shown in Table 1, and only the lowest values for each compound were considered in this evaluation. As expected, pepstatin A, a well-described and potent aspartyl peptidase inhibitor [18], had the lowest docking scores in comparison with the other ligands (except ritonavir for TcRP-B). In both modeled domains, pepstatin A interacts through hydrogen bonding with Gln252B and the catalytic residues Asp238A and B (Fig. 2A, D). Besides that, in the case of TcRP-A, pepstatin makes a hydrogen bond with Arg279A, Arg253A and B, and Gly250A, while with TcRP-B, only one hydrogen bond with Asp312A is observed. Considering the HIV-PIs, ritonavir and lopinavir had lower docking scores to TcRPA and B (Table 1), it is conceivable that they have the potential to inhibit the activity of both domains. As observed for the positive control, these inhibitors form hydrogen bonds with the catalytic aspartates (Fig. 2B, C, E, F). On the other hand, atazanavir and benznidazole were the worst ranked, presenting higher docking scores due mainly to the poor interactions observed (Fig. 2G–J). These two compounds probably have no inhibitory activity or binding on the aspartyl peptidase domains of DDI-1 like protein from *T. cruzi*.

**Table 1:** Molecular docking simulations between the 3D-modelled DDI1-like proteins from *T. cruzi* and HIV-PIs

TcRP-A		TcRP-B		HIV-1 peptidase	
Compounds	DS	Compounds	DS	Compounds	DS
1. Pepstatin A	− 9.526	1. Ritonavir	− 8.244	1. Saquinavir	− 12.086
2. Ritonavir	− 7.667	2. Pepstatin A	− 7.898	2. Atazanavir	− 12.073
3. Lopinavir	− 7.157	3. Lopinavir	− 7.869	3. Nelfinavir	− 10.810
4. Saquinavir	− 6.557	4. Nelfinavir	− 6.624	4. Tipranavir	− 10.573
5. Indinavir	− 6.527	5. Darunavir	− 6.072	5. Lopinavir	− 10.369
6. Tipranavir	− 6.318	6. Saquinavir	− 5.213	6. Ritonavir	− 9.907
7. Amprenavir	− 6.115	7. Amprenavir	− 4.824	7. Indinavir	− 9.171
8. Nelfinavir	− 6.076	8. Indinavir	− 4.781	8. Darunavir	− 8.874
9. Darunavir	− 5.729	9. Tipranavir	− 4.673	9. Amprenavir	− 8.764
10. Benznidazole	− 4.147	10. Atazanavir	− 4.5	10. Pepstatin A	− 8.388
11. Atazanavir	− 1.664	11. Benznidazole	− 3.662	11. Benznidazole	− 5.343

For comparison purposes, HIV aspartyl peptidase was also analyzed. Pepstatin A (positive control) and benznidazole (negative control)

TcRP-A and TcRP-B are 3D models of *T. cruzi* DDI1-like proteins, Tc00.1047053510155.40 and Tc00.1047053511585.40, respectively

*DS* docking score

The structures of HIV and *T. cruzi* aspartyl peptidases present remarkable 3D similarities, to verify if the molecular docking outcomes are also paralleled, docking experiments were carried out using HIV peptidase. Although the docking scores of ligands in different proteins cannot be directly compared, the use of positive and negative controls allows the comparison of binding modes and intermolecular interactions. Therefore, we used benznidazole again as a negative control and all the know HIV aspartic peptidase inhibitors as positive controls, including pepstatin A, which is also described in the literature as an inhibitor of the HIV enzyme [25]. As expected, benznidazole had the worst docking score, followed by pepstatin A. The top scored compounds were saquinavir (originally complexed in the studied HIV protein structure, presenting an RMSD of 1.03 Å in relation to the docking pose), atazanavir and nelfinavir. This result is in contrast to the *T. cruzi* protein that had pepstatin A, ritonavir and lopinavir as the better scored compounds (Table 1 and Additional file 1: Fig. S1). This contrast is expected since there was no rational design towards *T. cruzi* molecule. When comparing the predicted binding modes of these top scored inhibitors, similar key interactions can be observed in HIV and *T. cruzi* proteins, mainly with backbone and side chains of aspartates (including the catalytic ones) and glycines present in both active sites: Asp130B, Asp129B, Asp29A, Asp25A, Gly27A and Gly148B from HIV and Asp248A, Asp248B, Asp312A and Gly250A from *T. cruzi* (compare Fig. 2 and Additional file 1: Fig. S1). Therefore, the docking results predicted similar binding mechanisms, suggesting the binding of HIV PIs to *T. cruzi* DDI-1 protein in vitro.

DDI-1 gene product of *T. cruzi*, which shares homology to HIV aspartyl peptidase, is not biochemically characterized and has no crystallography structure resolved yet. In HIV and several fungi, HIV PIs target is an acidic aspartyl peptidase [26]. Curiously, although the intracellular target in trypanosomatids is still unknown, HIV PIs have a strong trypanocidal effect [3, 13–17], and have also a strong leishmanicidal activity [reviewed in 26]. The HIV PIs are capable of inhibiting the enzymatic cleavage of cathepsin D fluorogenic peptide substrates by crude extracts of either *T. cruzi* or *Leishmania* spp. [12, 14, 27]. To evaluate the structure–function relationship and the possible binding of HIV PIs to *T. cruzi* aspartyl peptidase, we realized homology modeling and molecular docking studies of the catalytic domain of the DDI-1 like protein from *T. cruzi*. The 3D model generated exhibited structural similarities with the proteins from the A2 aspartyl peptidase family, suggesting that the retroviral domain of the DDI-1 like must be functional. Molecular docking revealed that eight out of nine HIV IPs tested presented binding affinity similar to the positive control. The intracellular target of HIV IPs in *T. cruzi* is still unknown and multifactorial effects exerted by these inhibitors may be responsible for disturbance in parasite homeostasis that culminates in cell death. Recently, we have demonstrated on *Leishmania amazonensis* that one of the possible mechanisms of action of HIV IPs is related to lipids metabolism disturbance [27, 28]. Therefore, studies aiming to demonstrate the direct binding of HIV IPs in vitro to the DDI-1 like protein from *T. cruzi* can provide further information on the intracellular target of these inhibitors.

## Limitations

An important step towards the determination of the intracellular target of the HIV PIs in *T. cruzi* would be the direct demonstration of the binding or the aspartyl peptidase activity inhibition by these compounds towards the purified enzyme. Up to now, our attempts to generate active recombinant *T. cruzi* DDI-1-like aspartyl peptidase in *Escherichia coli* BL-21 or to purify the enzyme from *T. cruzi* crude extracts through pepstatin-A affinity chromatography were unsuccessful.

## Additional file

**Additional file 1: Figure S1**. Intermolecular interactions obtained from the molecular docking pose of the assayed compounds and HIV aspartyl peptidase. We selected three compounds with the highest hits from the molecular docking simulation: saquinavir, atazanavir, and nefilnavir (**A–C**). In **A**, the picture on the square represents the superposition of co-crystal (green) and redocked saquinavir structures (orange).

## Abbreviations

AIDS: acquired immune deficiency syndrome
DDI-1 like: DNA-damage inducible proteins
HAART: highly active anti-retroviral therapy
HIV IPs: HIV aspartic peptidase inhibitors
LigPrep: ligand preparation module
NTDs: neglected tropical diseases
PDB: Protein Data Bank
RMSD: root mean square deviation
