# Supplementary figures and images for: Docking simulation between HIV peptidase inhibitors and Trypanosoma cruzi aspartyl peptidase
Source: BMC Res Notes. 2018 Nov 21;11:825. doi: 10.1186/s13104-018-3927-z (PMC6249910; doi:10.1186/s13104-018-3927-z)

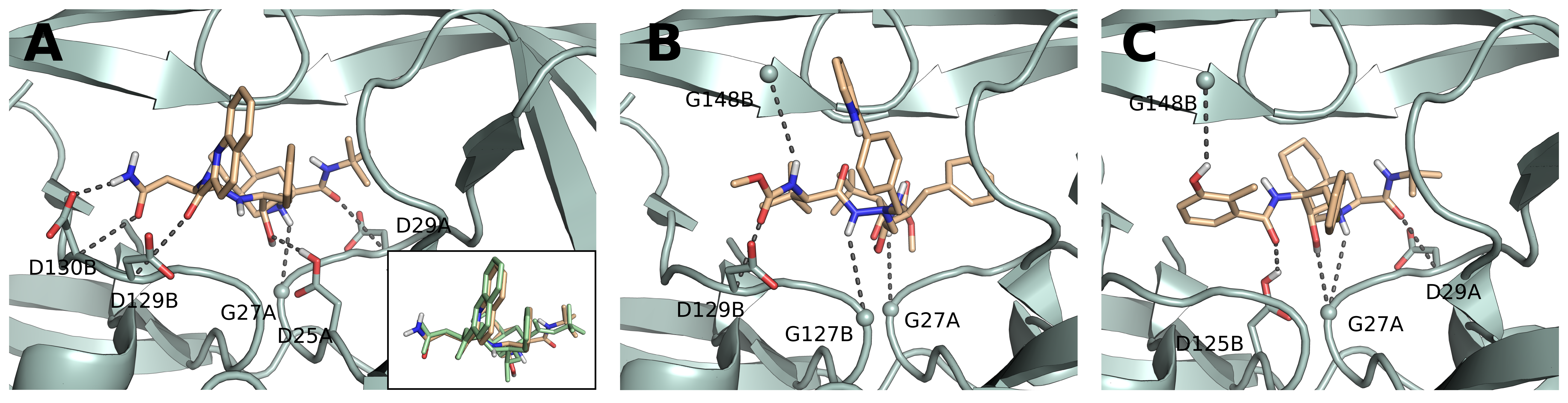

Supplement: Supplementary file 1 — Additional file 1: Figure S1. Intermolecular interactions obtained from the molecular docking pose of the assayed compounds and HIV aspartyl peptidase. We selected three compounds with the highest hits from the molecular docking simulation: saquinavir, atazanavir, and nefilnavir (A–C). In A, the picture on the square represents the superposition of co-crystal (green) and redocked saquinavir structures (orange). [file 13104_2018_3927_MOESM1_ESM.png]
